# The Effect of 1-MCP on the Expression of Carotenoid, Chlorophyll Degradation, and Ethylene Response Factors in ‘Qihong’ Kiwifruit

**DOI:** 10.3390/foods10123017

**Published:** 2021-12-05

**Authors:** Yanfei Liu, Guowen Lv, Jiaxin Chai, Yaqi Yang, Fengwang Ma, Zhande Liu

**Affiliations:** 1 College of Horticulture, Northwest A&F University, Xianyang 712100, China; lyfkiwi@nwafu.edu.cn (Y.L.); lvgw@nwafu.edu.cn (G.L.); jxchai@nwafu.edu.cn (J.C.); yaqiyang98@nwafu.edu.cn (Y.Y.); fwm64@nwsuaf.edu.cn (F.M.); 2 College of Life Science, Northwest A&F University, Xianyang 712100, China

**Keywords:** kiwifruit, beta-carotene, chlorophyll degradation, 1-MCP, eating ripe stage

## Abstract

The development of yellow color is an important aspect of fruit quality in yellow fleshed kiwifruit during fruit ripening, and it has a large influence on consumer preference. The yellow color is determined by carotenoid accumulation and chlorophyll degradation and is likely affected by ethylene production. This study investigates the expression of carotenoid, chlorophyll degradation, and ethylene response factors in ‘Qihong’ fruit, which had reached the near ripening stage (firmness ≈ 20 N) and were either left untreated (controls) or treated with 0.5 μL L^−1^ of 1-MCP for 12 h. Both the accumulation of *β*-carotene (not lutein) and degradation of chlorophyll *a* and *b* increased in response to the 1-MCP treatment, resulting in more yellow colored flesh in the 1-MCP treated fruit with higher carotenoid and lower chlorophyll contents. 1-MCP up-regulated *AcLCY-β*, *AcSGR1*, and *AcPAO2*, but reduced the expression of *AcCCD1*. These four genes were correlated with the concentrations of *β*-carotene and the chlorophylls. The expression of three ethylene response factors, including *Acc29730*, *Acc25620*, and *Acc23763* were delayed and down-regulated in 1-MCP treated fruit, showing the highest correlation with the expression of *AcLCY-β*, *AcSGR1*, *AcPAO2*, and *AcCCD1*. Dual-Luciferase assays showed that 1-MCP treatment not only eliminated the inhibition of *Acc23763* on the promoters of both *AcPAO2* and *AcLCY-β*, but also reduced the activation of *Acc29730* and *Acc25620* on the *AcCCD1* promoter. Our findings indicate that *Acc29730*, *Acc25620*, and *Acc23763* may play an important role in the response to 1-MCP treatment during the fruit eating ripe stage, which likely altered the promoter activities of carotenoid and chlorophyll-related genes (*AcPAO2*, *AcLCY-β* and *AcCCD1*) to regulate their transcripts, resulting in more yellow color in the fruit flesh of ‘Qihong’.

## 1. Introduction

Kiwifruit (*Actinidia chinensis*) is known as the ‘king of fruit’ because of its unique flavor and exceptional nutritional value (e.g., vitamin C), and has become one of the most recently developed and economically important fruit crops worldwide [1]. Yellow fleshed kiwifruit has become more widely preferred by consumers due to its brilliant color and rich carotenoids, compared with their green-fleshed cousins. Chlorophyll degradation and β-carotene accumulation are associated with changes in flesh color, and both kinds of pigments are responsible for the development of yellow color in kiwifruit during ripening [2,3,4].

Chlorophyll degradation is an important component of the de-greening process necessary for marketability of most yellow climacteric fruit during ripening (e.g., citrus, papaya, and banana) [5,6,7,8], and thus has become a special focus for an increasing number of researchers. It has been previously shown that ethylene is involved in chlorophyll degradation in fruit. For example, exogenous ethylene treatment accelerates chlorophyll degradation in mature green banana, citrus, and avocado fruit [9,10,11]. Treatment with the ethylene antagonist 1-methylcyclopropene (1-MCP) leads to retention of the green color in fruit by reducing chlorophyllase activity [6,12,13,14]. 

Ethylene also regulates carotenoid biosynthesis during fruit maturation. Hoeberichts et al. reported that ethylene treatment stimulates the *PSY* transcript level in tomato fruit [15]. Marty et al. suggested that applying ethylene to mature green apricots postharvest increases expression of the *PSY* and *PDS* genes, resulting in the accumulation of phytofluene and phytoene [16]. In durian fruit, ethylene affected the carotenoid accumulation by regulating the gene expression in the carotenoid pathway, including zeta-carotene desaturase (*ZDS*), lycopene beta-cyclase (*LCYB*), chromoplast specific lycopene beta-cyclase (*CYCB*) and beta-carotene hydroxylase (*BCH*) [17]. 1-MCP treatment inhibits carotenoid biosynthesis in some fruit. However, this depends on the carotenoid compounds and whether the fruit can be induced by endogenous ethylene during ripening [16,18,19]. Carotenoid accumulation decreases in response to 1-MCP treatment of papaya fruit, compared to untreated control fruit [20]. 1-MCP applied before ripening also reduces carotenoid synthesis by inhibiting transcript levels of the *PSY*, *ZDS*, *LCYB*, and *BCH* genes in nectarines [21]. A slight inhibition of carotenoid accumulation and *PSY* expression were detected in 1-MCP-treated apricot [18].

Kiwifruit are classified as a climacteric fruit [22], and four distinct softening phases were clearly described during postharvest ripening [23]. Fruit at phase 1 and phase 2 did not produce endogenous ethylene but were highly sensitive to exogenous ethylene, and many fruit quality characters (e.g., color, flavor, and taste) were affected by the application of exogenous ethylene [18,24,25,26,27,28]. When fruit firmness decreased to about 20% of their harvest value (phase 3), fruit was considered to be in the eating ripe stage for consumers. At this stage, fruit started to produce autocatalytic ethylene, and about one week after, fruit became over-ripe and senescence (phase 4). Richardson et al. suggested that the color change of ‘Hort16A’ finished at stage 85, when the outer pericarp turned yellow with a 100° hue angle [29]. But in fact, the final hue value of ‘Hort16A’ is ~97° in fully ripened fruit [30], indicating the flesh yellow color change may still occur at the phase 3 stage, and it is also an important eating quality character for consumers. However, the changes in carotenoids and chlorophylls and the transcript of related genes during this stage are not fully clear.

**Figure 1:**
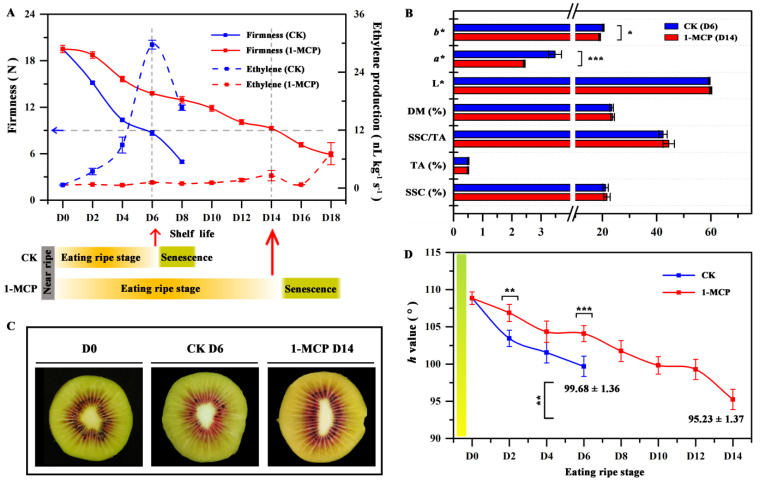
The fruit physiology indicators and flesh color after 1-MCP treatment. (**A**) fruit firmness and ethylene production; (**B**) SSC, TA, DM, and color of kiwifruit after 1-MCP treatment; (**C**) the phenotype of control fruit at D6 and 1-MCP treated fruit at D14; (**D**) h value during the eating ripe stage. Data are mean ± SE (*n* = 3). Values with different letters represent significant differences in the same group (1-MCP group or control group) during ripening (*p* < 0.05). Asterisk represents a significant difference between the control (CK) and 1-MCP treated fruit (* *p* < 0.05, ** *p* < 0.01 and *** *p* < 0.001).

In our previous study, we found the color indexes a* and b* of ‘Qihong’ (yellow outer pericarp and red inner pericarp) were significantly changed during their eating ripe window with firmness ≈ 20 N to 8 N [31]. It was noteworthy that, similar to the loss of firmness, the color changes were obviously affected by 1-MCP treatment [31], which greatly delayed and reduced the production of autocatalytic ethylene and resulted in the stronger yellow color and lower hue angle (Figure 1A). 

Therefore, this study investigates the effect of 1-MCP on the flesh yellow color change in ‘Qihong’ during the eating-ripe stage. This includes the crucial changes in the concentrations of chlorophylls and carotenoids and the transcript profiles of genes related to pigment metabolism and ethylene response factors (ERFs). The final aim of our study was to reveal the correlation between ethylene and yellow color changes in ‘Qihong’ fruit and to provide a theoretical basis for improving their eating quality.

## 2. Materials and Methods

### 2.1. Fruit Materials and Treatment

*Actinidia chinensis* cv. ‘Qihong’ fruit sampled from a commercial orchard in Shannxi Province, China, in 2019 was used in this study. The harvested fruit (firmness 100.74 N, SSC 13.9%, DM 19.8%) was stored at room temperature (23 ± 1 °C) until they reached near eating firmness (20 N, 60 fruits were used to determine the eating firmness), uniform fruit without visible wound signals were selected for further treatment. About 600 fruit were divided into control (CK) and 1-MCP groups. The control group without any treatment was placed directly into a 90 L sealed container for 12 h at 23 °C. The second group was treated with 0.5 μL L^−1^ 1-MCP (SmartFresh^®^, AgroFresh, Spring House, Philadelphia, PA, USA) in the same size sealed container for 12 h, 23 °C. Subsequently, the two groups of kiwifruit were transferred to a storage room at 23 °C [31]. At each sampling point, 27 fruit were collected from each group and divided into three replicates. The outer pericarp was immediately frozen in liquid nitrogen and stored at −80 °C pending analysis.

### 2.2. Measurement of Physiological Indices

About 1 mm thick and 1 cm^2^ peel at the two opposite cheeks of each fruit were removed first, then the GS-15 Fruit Texture Analyzer (Strand, Cape Town, South Africa) with an 8 mm plunger was used to determine the fruit firmness [32]. Two juice samples from the equatorial part of each fruit were used to measure the soluble solid content (SSC) and titratable acidity (TA) with the PAL-1 (Atago, Tokyo, Japan). A slice sampled from the fruit equator was used to measure dry matter content. The transverse fruit slices (2 mm) were weighed to record their fresh weights (FW) and were placed at 75 °C (DZF-6050, Shanghai, China) for 24 h (DW). Dry matter content was determined by: FW/DW × 100% [33]. Flesh color (L*, a* and b*) was measured using a CR-400 chromameter (Konica Minolta, Tokyo, Japan) [34]. Three replicates were carried out for these physiological indices.

### 2.3. Carotenoid and Chlorophyll Measurements

The chlorophylls and carotenoids were extracted from the kiwifruit according to previous methods [3,4]. The extracts were quantified using a high performance liquid chromatography (HPLC) system (1260 Infinity II, Agilent Technologies, Palo Alto, CA, USA) equipped with a DAD detector and a C-18 column (250 nm × 4.6 mm, 5.0 μm, GL Sciences Inc., Tokyo, Japan). The injection volume was 20 μL, the post-run time was 5 min, and the flow rate was 0.7 mL min^−1^ at 40 °C. The solvent consisted of 90% (*v*/*v*) acetonitrile/water (A) and ethyl acetate (B). The gradient profile was 100% A (0 min), 20% A (14 min), 20% A (20 min), and 100% A (30 min). Chlorophyll *a* was simultaneously monitored at 430 nm, while lutein, β-carotene, and chlorophyll *b* were monitored at 450 nm. 

### 2.4. Gene Identification and Quantitative Polymerase Chain Reaction (qPCR) Analysis

All gene sequences to be tested were obtained from the kiwifruit genome database as a reference [35,36]. The transcriptome data (PRJNA277383) was used to analyze the responses of the AcERFs to ethylene treatment and the results are shown as a heatmap. Total RNA was extracted using the Plant RNA Kit (Omega Bio-tek, Norcross, GA, USA). Then, approximately 1 μg of total RNA was used for cDNA synthesis with the Prime-Script RT reagent kit (TaKaRa, Dalian, China). Quantitative real-time PCR (20 μL) was monitored on the LightCycler 96 system (Roche, Basel, Switzerland) with the SYBR Premix ExTaq II Kit (TaKaRa). The amplification program was 98 °C for 30 s for one cycle, 95 °C for 5 s for 40 cycles, and 57 °C for 30 s. Gene expression levels were normalized with the 2^−ΔΔCT^ method [37] using the *AcActin* and *AcPP2A* genes as internal standards. All analyses were repeated three times using biological replicates. The specific primers are listed in Table S1.

### 2.5. Correlation Analysis and Network Visualization

A correlation analysis was performed between the contents of *β*-carotene, chlorophyll *b*, and chlorophyll *a*, and the expression of all tested genes was carried out using OmicShare tools (http://www.omicshare.com/tools). Results were visualized as a network using Cytoscape (v3.7.1).

### 2.6. Promoter Analysis

Using the kiwifruit genome database as a reference [34,35], the promoter sequences of *AcPAO2*, *AcSGR1*, *AcLCY-β*, and *AcCCD1* were isolated. Newplace was used to analyze potential ERF binding motifs [38].

### 2.7. Dual-Luciferase Assays

According to previous reports [39,40], the promoter sequences were cloned into the pGreenII 0800-LUC vector forming the reporters, the full CDS were cloned into the pGreenII 62-SK and used as the effectors (Table S1, Figure S3). The *A. tumefaciens* strain GV3101- pSoup were used to introduce the fused constructs. The cultures with OD_600_ of 0.8 were infiltrated with buffer (10 mM MgCl_2_, 10 mM MES, 200 mM acetosyringone and pH 5.8). The different *Agrobacterium* mixtures were injected into the *N. benthamiana* leaves of 6-week-old plants. Then, tested tobaccos were classed into two groups—control (without any treatment) and 1-MCP treatment (0.1 μL L^−1^, 2 h, 23 °C). After 48 h, according to the manufacturer’s instructions, the Dual-Luciferase Reporter Assay System (Promega, Madison, WI, USA) was used to detected the luciferase activity.

### 2.8. Statistical Analysis

The results were analyzed by one-way analysis of variance in SPSS 22.0 software (SPSS Inc., Chicago, IL, USA) to compare significant differences among treatments, using Duncan’s multiple range test (*p* < 0.05) and Student’s *t*-test (* *p* < 0.05, ** *p* < 0.01 and *** *p* < 0.001). All results are presented as mean ± standard error.

## 3. Results and Discussion

### 3.1. The Fruit Physiology Indicators and Flesh Color after 1-MCP Treatment

The firmness of climacteric fruit is influenced by the concentrations and exposure duration to exogenous and endogenous ethylene [7]. Based on our published report [31], profiles of firmness and ethylene production were further compared between 1-MCP treated and untreated groups (see Figure 1A). 1-MCP significantly delayed and reduced the peaks of the ethylene production compared with the control fruit during storage at 23 ± 1 °C and 90–95% RH. The firmness of control fruit declined until day 8 (D8), reaching 8.65 ± 0.30 N at D6, which is the optimum period for eating (best flavor, taste, and texture) [29]. As expected, the 1-MCP treatment reduced the softening rate, and firmness declined to the key value at D14 with firmness 9.27 ± 0.23 N, resulting in a longer shelf life, which was similar to a previous study [25]. The combined ethylene production and firmness change meant that stage D6 and D14, respectively, identified the final stage of the eating ripe window in control and 1-MCP treated fruit (Figure 1A). No differences (*p* < 0.05) in SSC, TA, SSC/TA, or DM and L*, were found between the control (D6) and 1-MCP treated fruit (D14). However, the flesh of the 1-MCP treated fruit at D14 was more yellow in color (lower a*, b* value and *h* value) than that of the control fruit at D6 (Figure 1B–D), indicating that the 1-MCP treatment led to higher exterior quality of ’Qihong’ fruit.

### 3.2. The Fruit Carotenoid Contents in 1-MCP Treated Fruit and Control Fruit

Carotenoid, including lutein and *β*-carotene, are the pigments responsible for flesh coloration in yellow fleshed kiwifruit, and *β*-carotene is mainly responsible for the gold-yellow color of kiwifruit [3,4].

**Figure 2:**
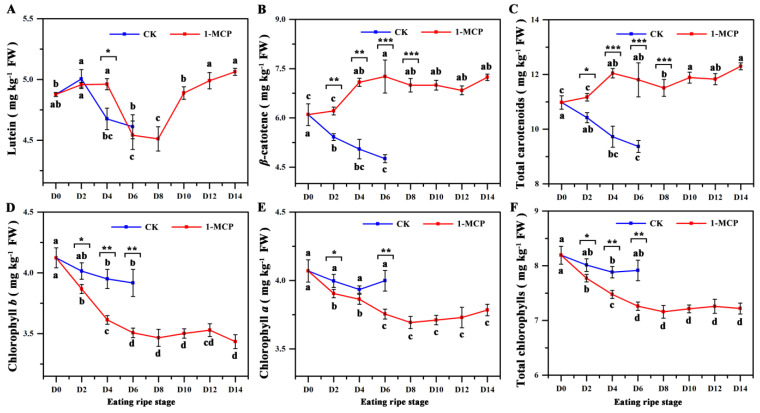
The contents of carotenoid and chlorophyll in treated fruit as measured by HPLC. *β*-Carotene (**A**), lutein (**B**), total carotenoids (**C**), chlorophylls *b* and *a* (**D**,**E**), and total chlorophylls (**F**). Data are mean ± SE (*n* = 3). Different letters represent significant differences in the same group during ripening (*p* < 0.05). Asterisk represents a significant difference between the control (CK) and 1-MCP treated fruit (* *p* < 0.05, ** *p* < 0.01, *** *p* < 0.001).

Among the quantified carotenoid of the two types, the concentration of lutein decreased in both the control and 1-MCP treated groups before D6, then increased in the 1-MCP treatment groups (Figure 2A). *β*-carotene decreased gradually in the control fruit during eating ripe window; while in 1-MCP treated fruit, it rapidly increased and reached a peak on D6, then was relatively smooth until D14 (Figure 2B). The *β*-carotene content in the 1-MCP treated fruit (7.24 ± 0.10 mg kg^−1^ FW) was higher during the final stage than that in the control (only 4.76 ± 0.12 mg kg^−1^ FW). Thus, the 0.5 μL L^−1^ 1-MCP treatment may have delayed kiwifruit senescence and led to normal *β*-carotene biosynthesis. Our findings differ from those of previous reports [17,20], who suggested that 1-MCP treatment delays the accumulation of carotenoid due to the time difference in the 1-MCP application. Both studies applied 1-MCP before fruit ripening at a time when the fruit had not finished post-ripening metabolic processes, while our study applied 1-MCP near the eating period (initiation of phase 3) when the fruit had basically finished post-ripening (phase 1 and 2), such as starch degradation, rapid accumulation of SSC, and chlorophyll degradation.

### 3.3. The Fruit Chlorophyll Contents in 1-MCP Treated and Control Fruit

Chlorophyll degradation is an important trait that accompanies fruit ripening in most species [6]. Exogenous ethylene accelerates the degradation of chlorophylls in mature green citrus and avocado fruit [9,10,11]. Exogenous 1-MCP applied to citrus, banana, apple, and pear inhibits fruit softening and delays degradation of chlorophylls during fruit ripening [6,14,41,42]. Interestingly in this study, as shown in Figure 2D–F, the 1-MCP treatment sped up the decrease in chlorophyll *b*, chlorophyll *a*, and chlorophyll *a* + *b* content, while there was a small difference in chlorophyll contents through all of the sampled days in untreated fruit. The contents of chlorophyll *b*, *a*, and *a* + *b*, in treated fruit during the best eating period (the final stages) were 3.42 ± 0.06, 3.78 ± 0.01, and 7.20 ± 0.08 mg kg^−1^ FW, respectively, which was 12.35, 5.36, and 7.67% lower than that in untreated fruit. These findings seem inconsistent with previous reports [14,25,41]. 

To better explain this conflict, the reports of Xu et al. [25] and Lv et al. [14] were further investigated. Xu et al. suggested that, in green kiwifruit ‘Hayward’, 1-MCP treatment inhibited the process of chlorophyll degradation [25]. It was noteworthy that the chlorophyll content in untreated fruit was approximately 6.9 g kg^−1^ (Figure S1), which was obviously higher than that in 1-MCP treated fruit, which was about 4.5 g kg^−1^ at the best eating ripe stage (firmness ~9 N) [25]. The same result has also been found in apple fruit; it exhibited lower chlorophyll content and hue angle (H°) at 49 days (ethylene peak) compared to untreated fruit at 28 days (Figure S2), though 1-MCP delayed the degradation of chlorophyll during ripening [14].

### 3.4. Expression of Genes Involved in Carotenoid Biosynthesis and Degradation 

**Figure 3:**
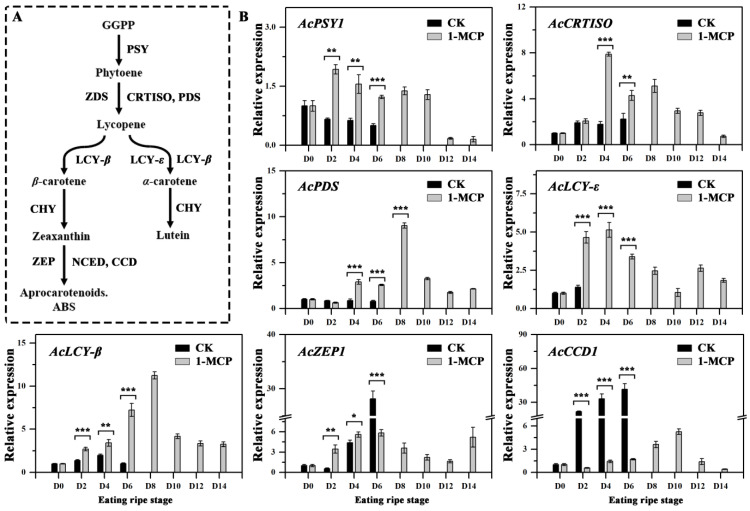
Expression profiles of genes involved in the carotenoid pathway in the flesh of kiwifruit. (**A**) Carotenoid biosynthetic and degradation pathways in kiwifruit. (**B**) Effect of 1-MCP on the expression profiles of carotenoid pathway related genes in the flesh during storage. The D0 stage was used as the reference (=1 relative expression) for each gene. Error bars show SE of the mean. Data were analyzed with Student’s *t*-test (* *p* < 0.05, ** *p* < 0.01, *** *p* < 0.001).

The carotenoid metabolic pathway has been studied in many fruit species [17], including kiwifruit, as shown in Figure 3A [2,35]. The expression of carotenoid metabolic pathway genes was investigated in the untreated and treated groups. *AcZDS*, *AcCHYs*, and *AcNCED*s expression was not detected in any of the samples. The expression levels of *AcPSY1*, *AcCRTISO*, *AcPDS*, *AcLCY-ε*, and *AcLCY-β*, which are in the carotenoid biosynthetic pathway, were lower in control fruit than those in 1-MCP treated fruit, and gradually increased at the early stage but then decreased. The expression profiles of *AcZEP1* in the 1-MCP-treated fruit increased from D2 to D6 and decreased on D8 compared with the controls. *AcCCD1* is an important gene responsible for *β*-carotene degradation [3]. It increased in control fruit during storage, and maximum expression was detected on D6. This level was always higher than that in 1-MCP treated fruit. This finding indicated that the 1-MCP treatment inhibited the expression of *AcCCD1*.

### 3.5. Expression of Genes Involved in Chlorophyll Degradation

**Figure 4:**
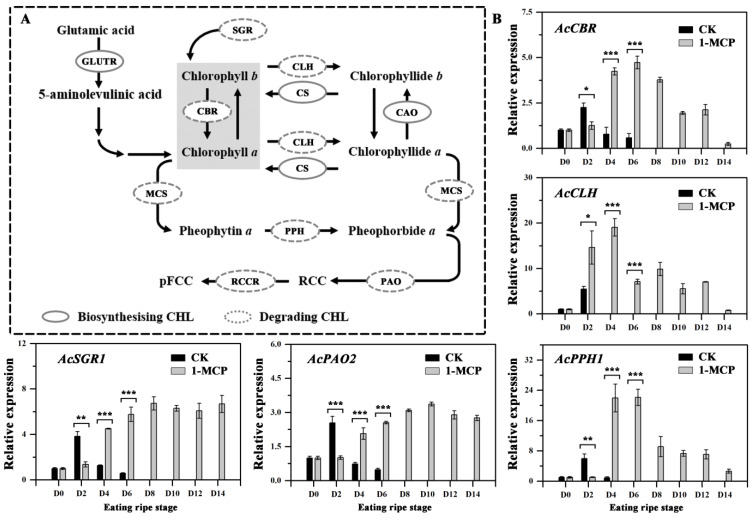
Expression profiles of genes involved in chlorophyll degradation in the flesh of kiwifruit. (**A**) Chlorophyll degradation pathway in kiwifruit. (**B**) Effect of 1-MCP on the expression profiles of chlorophyll degradation-related genes in the flesh during storage. The D0 stage was used as a reference (=1 relative expression) for each gene. Error bars show SE of the mean. Data were analyzed with Student’s *t*-test (* *p* < 0.05, ** *p* < 0.01, *** *p* < 0.001).

The nine genes related to chlorophyll degradation were selected (Table S1) and investigated during the post-treatment period (Figure 4). *AcSGR2*, *AcPAO1*, *AcPPH2*, and *AcRCCR* were not expressed in treated or untreated fruit. It has been shown that *ASGR1* and *AcPAO2* are the key genes responsible for chlorophyll degradation in kiwifruit [2,3]. In this study, the expression of both genes increased gradually and reached a peak on D2 but then decreased sharply in control fruit; while in 1-MCP treated fruit, the expression of them was higher than that in control fruit during storage except at the D2 stage. The expression levels of *AcCBR*, *AcCLH*, and *AcPPH1* changed similarly between the control and 1-MCP treated fruit during storage. Overall, our findings suggest that the effect of 1-MCP is to delay the increase in the expression of chlorophyll degradation genes within a short time, but it up-regulated their expression during later stages of storage.

### 3.6. Expression of Ethylene Related Genes in Kiwifruit during Storage

**Figure 5:**
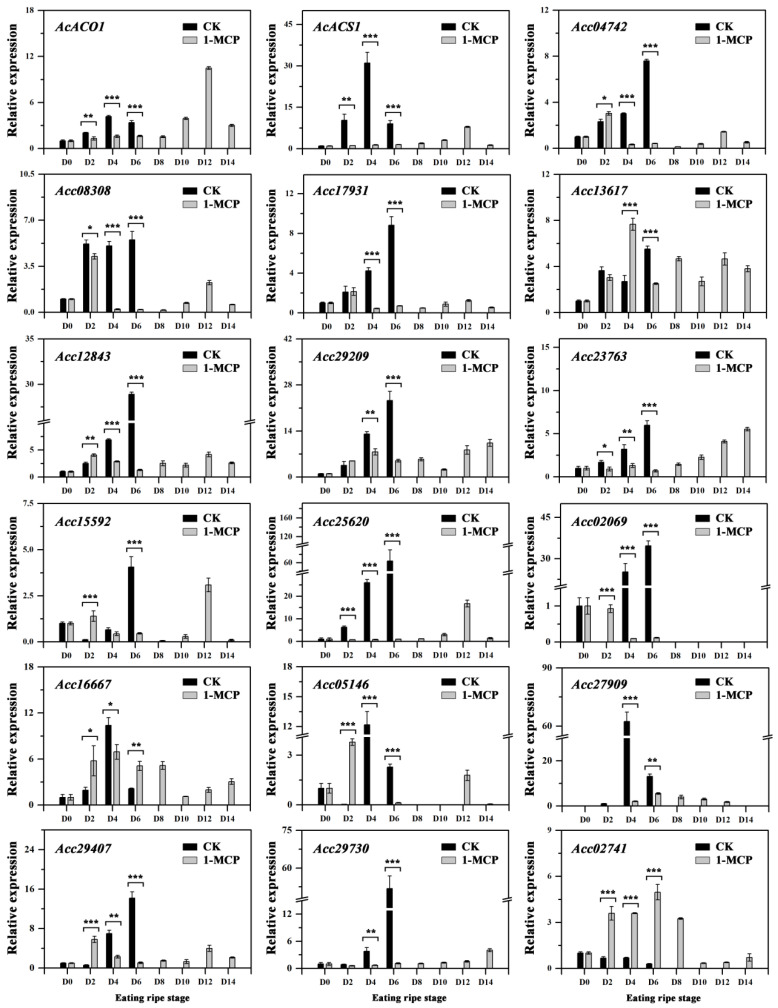
Expression of the ACO and ACS genes and ethylene-induced ERFs in the flesh of kiwifruit. Error bars show SE of the mean. Data were analyzed with Student’s *t*-test (* *p* < 0.05, ** *p* < 0.01, *** *p* < 0.001).

Our previous study showed that use of 1-MCP delayed the peaks of the ethylene production [31] (Figure 1A), thus it was supposed that ethylene may play an important role in the difference formation of ‘Qihong’ flesh color. ACO and ACS are the main ethylene producing enzymes in various fruit, such as tomato [43,44], apple [45], pear [46], and melon [47]. In kiwifruit, *AdACO1* and *AdACS1* have been functionally verified to be responsible for ethylene biosynthesis during fruit ripening [23,27]. As shown in Figure 5, *AcACO1* and *AcACS1* expression levels were similar during storage. Both genes increased from D0 and reached a peak on D4 then decreased in control fruit. The expression of these two genes in 1-MCP treated fruit was significantly lower than that in control fruit from D0 to D6. Expression then increased after this stage and reached a peak on D12. These findings agree with Wu et al. [27], who suggested that 1-MCP treatment delays *AdACO1* and *AdACS1* expression.

It is well-known that the AP2/ERF transcription factors (TFs) play an important role in the response to 1-MCP treatment [6]. In this study, 153 individual AcAP2/ERF TFs were found in the Red 5 genome. Among them, 18 ERFs, which were up-regulated by the ethylene treatment during storage (Figure S1), were investigated further by qPCR (Figure 5). *Acc22453* and *Acc00583* expression was not detected in treated or untreated fruit. *Acc04742*, *Acc08308*, and *Acc17931* were expressed at higher levels from D2 to D6 in control fruit than in 1-MCP treated fruit. *Acc12843* and *Acc29209* expression increased in control fruit during storage, and higher transcript levels were detected during D4–D6 than those in treated fruit. Notably, three genes (*Acc23763*, *Acc25620*, and *Acc29730*) increased continuously and reached peaks on D6 in the control fruit, while the transcript levels and the highest peaks (both abundance and time) of these genes were inhibited and delayed by the 1-MCP treatment. *Acc02741* was highly expressed in 1-MCP treated fruit from D2 to D8, and the level was significantly higher than that in control fruit. In contrast, the expression of other genes, including *Acc13617*, *Acc15592*, *Acc02069*, *Acc16667*, *Acc05146*, *Acc27909*, and *Acc29407* was complex and no significant pattern was found between the control and 1-MCP treated fruit.

### 3.7. Correlation Analysis of Pigment Contents and Expression of Related Genes

**Figure 6:**
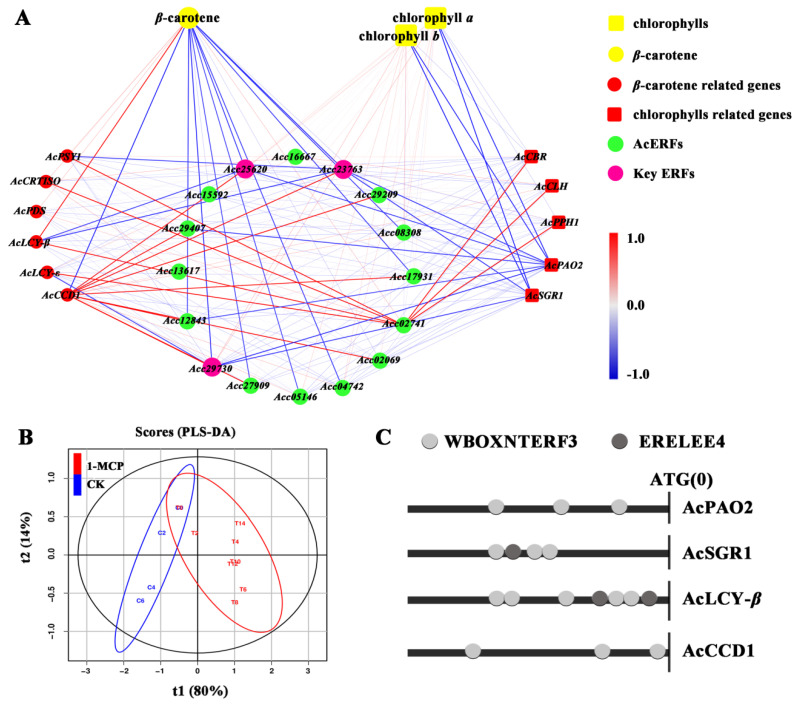
Correlation network and promoter analyses. (**A**) Correlation network analysis for pigment contents, pathway related genes, and the tested ERFs. Correlations are indicated by the colored bar. (**B**) The PLS-DA analysis for genes with significant correlations. (**C**) The promoter analysis of four important pathway related genes.

The correlations between the contents of *β*-carotene, chlorophyll *b*, and chlorophyll *a* and the expression of all tested genes were assessed and visualized using Cytoscape (v3.7.1). As shown in Figure 6A, *β*-carotene content was positively correlated with the *AcLCY-β* transcript changes, but was negatively correlated with the *AcCCD1* transcript. These results are consistent with previous studies suggesting that *AcLCY-β* and *AcCCD1* are responsible for *β*-carotene biosynthesis and degradation in kiwifruit, respectively [2,3]. Similar to the findings of these two reports, negative correlations were detected between chlorophyll contents and the *AcPAO2* and *AcSGR1* transcript levels. Furthermore, *Acc25620*, *Acc29730*, and *Acc23763* were negatively correlated with *AcLCY-β*, *AcPAO2*, and *AcSGR1*, but positively correlated with *AcCCD1*. A partial least-squares discriminant analysis (PLS-DA) showed that pigment contents and the expression of several genes in the samples were classified into the control and 1-MCP treatment groups (Figure 6B).

### 3.8. The Activation of AcSGR1, AcPAO2, AcLCY-β and AcCCD1 Promoters by Acc25620, Acc29730 and Acc23763 after 1-MCP Treatment

The ERF TFs have been reported to play important roles (either positive or negative) in plant growth and stress response [48,49]. In this study, all three ERFs contained the ERF motif, and an EAR motif also found in the C-terminal region of Acc23763 (Figure S2), indicating it may act as a repressor [48]. Moreover, at least three ERF binding motifs (WBOXNTERF3 or ERELEE4) [6] were found in the *AcPAO2*, AcSGR1, *AcLCY-β*, and *AcCCD1* promoters (Figure 6C).

**Figure 7:**
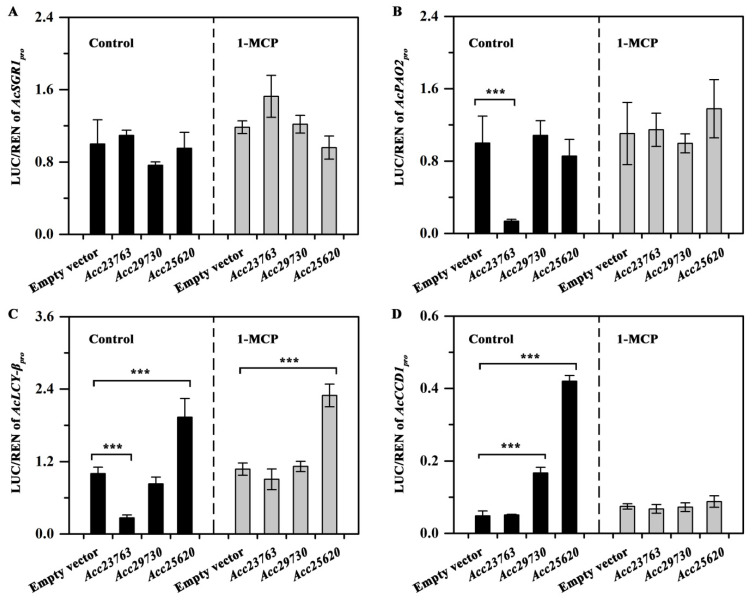
The activation of *AcSGR1* (**A**), *AcPAO2* (**B**), *AcLCY-β* (**C**) and *AcCCD1* (**D**) promoters by *Acc25620*, *Acc29730*, and *Acc23763* after 1-MCP treatment. Error bars show the standard errors of the means (*n* = 3). *** *p* < 0.001.

In citrus, *CitERF13* enhances activity of *CitPPH* by directly binding to its promoter, therefore accelerating chlorophyll degradation [6]. In this study, as shown in Figure 7A, the promoters of *AcSGR1* were not activated, in both control and 1-MCP treatment groups. *Acc23763* strongly reduced the promoters of both *AcPAO2* and *AcLCY-β*, however, this reduction was eliminated by 1-MCP treatment (Figure 7B, C). The promoter of *AcLCY-β* was activated by *Acc25620* in both control and 1-MCP groups. *Acc29730* and *Acc25620* strongly activated the *AcCCD1* promoter by approximately 3.46- and 8.72-fold; while it was noteworthy that 1-MCP treatment reduced above activation, no difference (*p* < 0.05) was found between the empty vector and *Acc29730* and *Acc25620* (Figure 7D).

## 4. Conclusions

In this study, we showed that 1-MCP treatment affected yellow color formation of flesh in ‘Qihong’, which affected carotenoid accumulation and chlorophyll degradation in kiwifruit. This effect was correlated with the expression of key pathway genes, such as *AcPAO2*, *AcSGR1*, *AcLCY-β*, and *AcCCD1*. The expression of three ERFs, including *Acc29730*, *Acc25620*, and *Acc23763* was delayed and down-regulated by the 1-MCP treatment, showing higher correlation with the expression of *AcLCY-β*, *AcSGR1*, *AcPAO2*, and *AcCCD1*. Dual-Luciferase assays showed that the inhibition of *Acc23763* on the promoters of both *AcPAO2* and *AcLCY-β*, and the reduction of the *Acc29730* and *Acc25620* on the *AcCCD1* promoter were obviously eliminated by 1-MCP treatment. In summary, the development of the yellow flesh color in ‘Qihong’ appears to be regulated by 1-MCP, which modulated the expression of *Acc25620*, *Acc29730*, and *Acc23763*. These ERFs affected the promoter activities of carotenoid and chlorophyll related genes (*AcPAO2*, *AcLCY-β* and *AcCCD1)* to regulate their transcript levels, leading to more yellow color in ‘Qihong’ fruit flesh with higher carotenoid content and lower chlorophylls content. However, this conclusion requires additional studies to verify. Overall, our study provides a theoretical basis for improving the eating quality of ‘Qihong’ fruit.

## Data Availability

The data presented in this study are available on request from the corresponding author. The data are not publicly available due to the data contains unpublished content from other articles.

## Supplementary Materials

The following are available online at https://www.mdpi.com/article/10.3390/foods10123017/s1, Figure S1: The firmness and chlorophyll content during storage in the report of Xu et al. 2019, Figure S2: The ethylene production, H value and chlorophyll content during storage in the report of Lv et al. 2020, Figure S3: Heatmap of AcERFs in kiwifruit during ripening. The genes signed by red boxes were used further studied, Figure S4: Amino acid sequence alignments of three AcERFs, Figure S5: Schematic diagrams used for the dual-luciferase assay, Table S1: The primers used in this study.
