# Supplementary figures and images for: The Effect of 1-MCP on the Expression of Carotenoid, Chlorophyll Degradation, and Ethylene Response Factors in ‘Qihong’ Kiwifruit
Source: Foods. 2021 Dec 5;10(12):3017. doi: 10.3390/foods10123017 (PMC8701096; doi:10.3390/foods10123017)

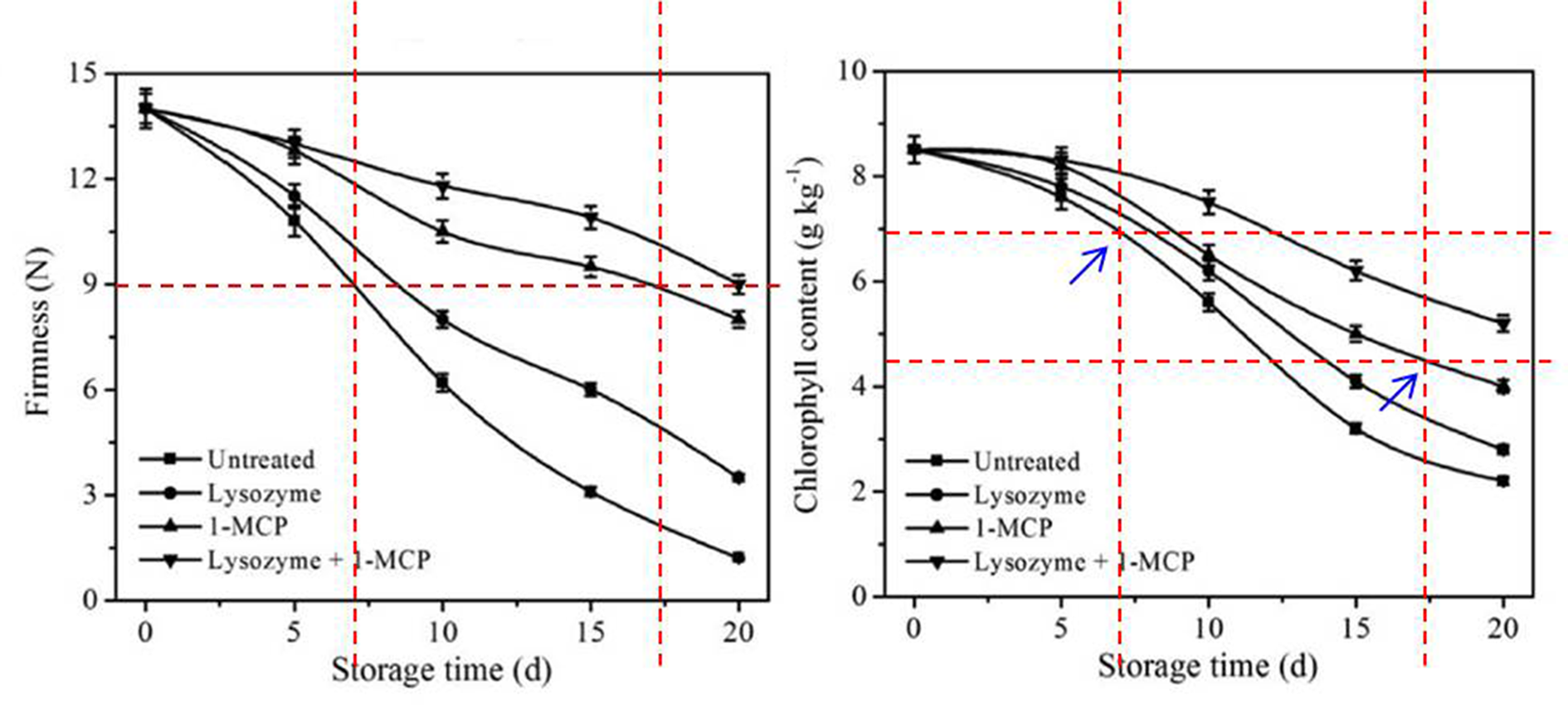

Supplement: Supplementary file 1 [file foods-10-03017-s001.zip › Figure S1.tif]

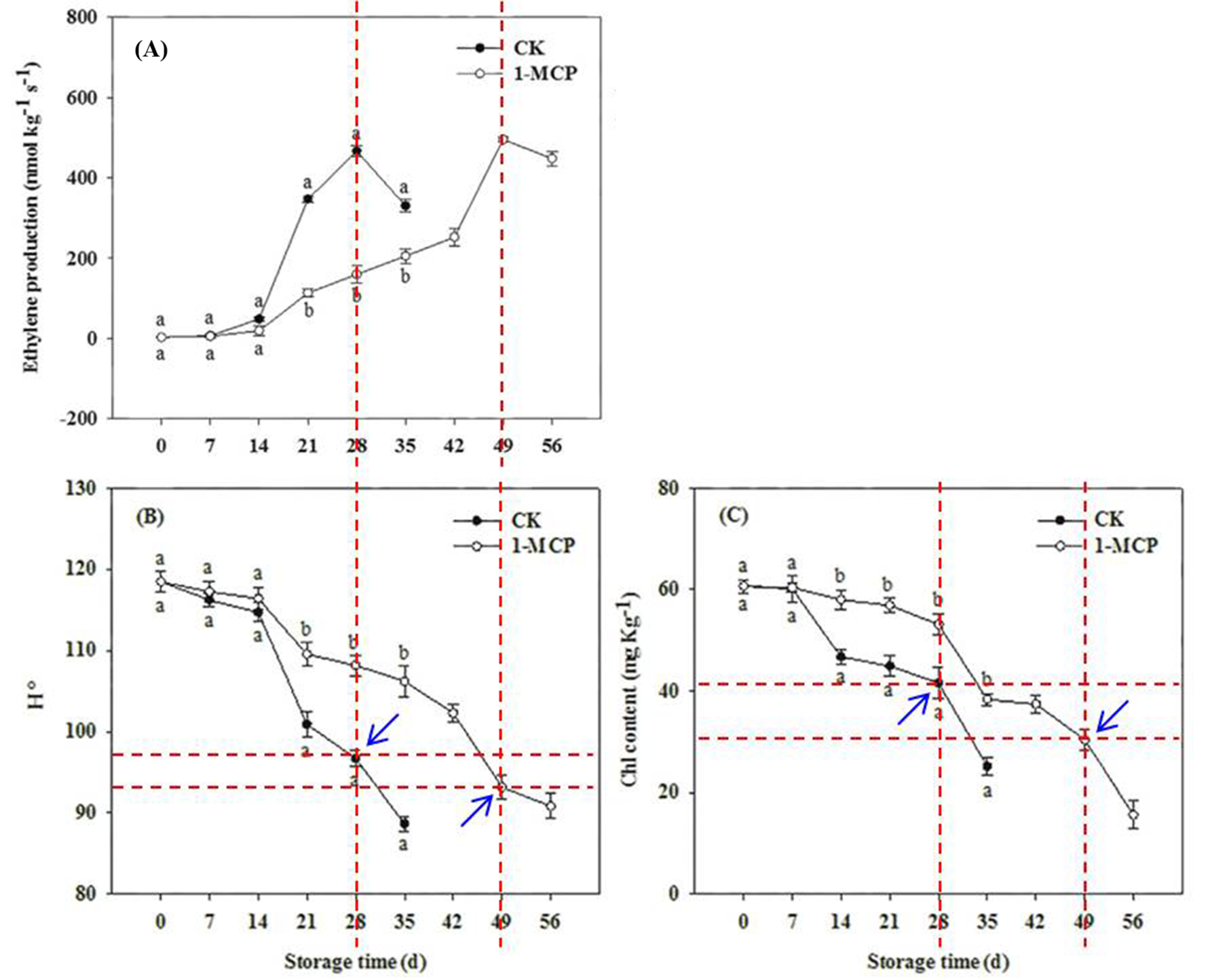

Supplement: Supplementary file 1 [file foods-10-03017-s001.zip › Figure S2.tif]

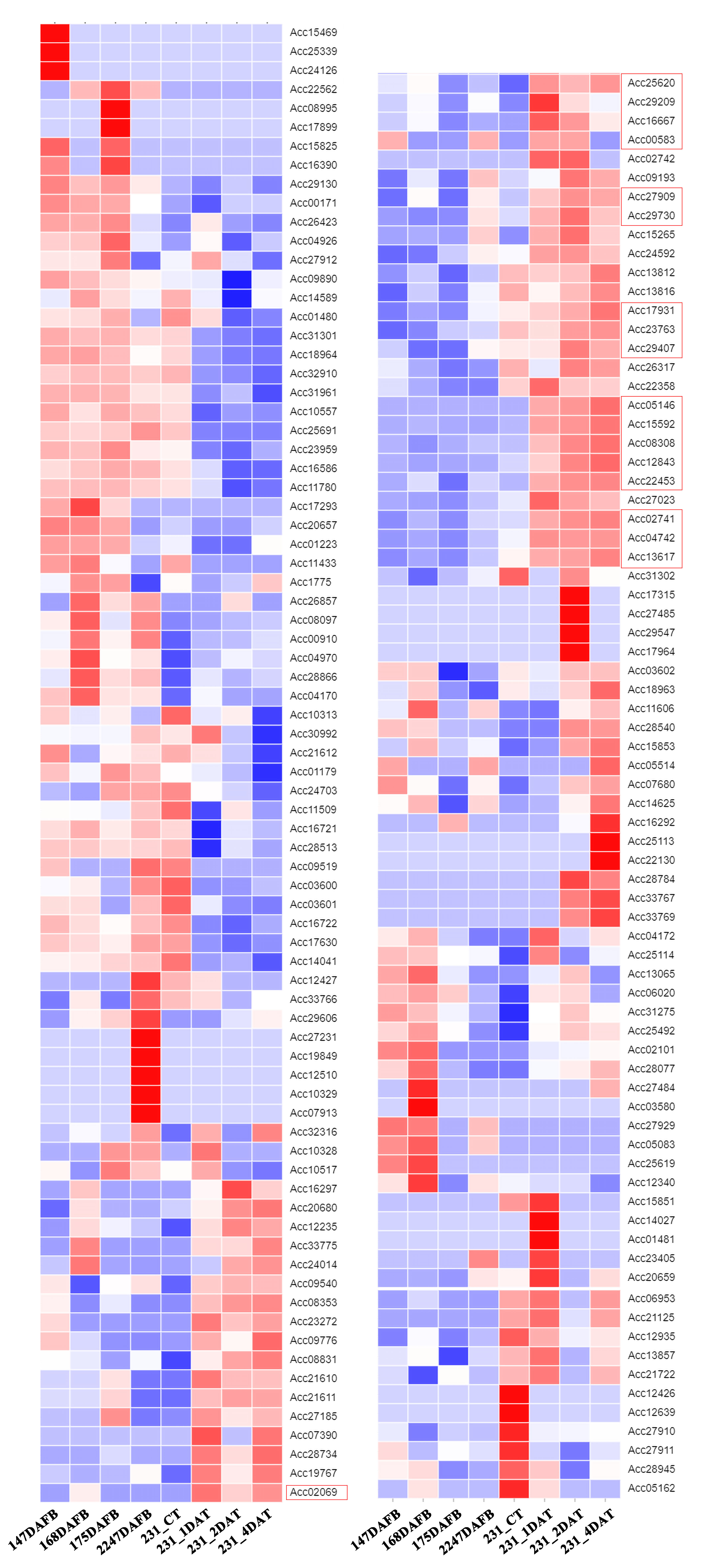

Supplement: Supplementary file 1 [file foods-10-03017-s001.zip › Figure S3 .tif]

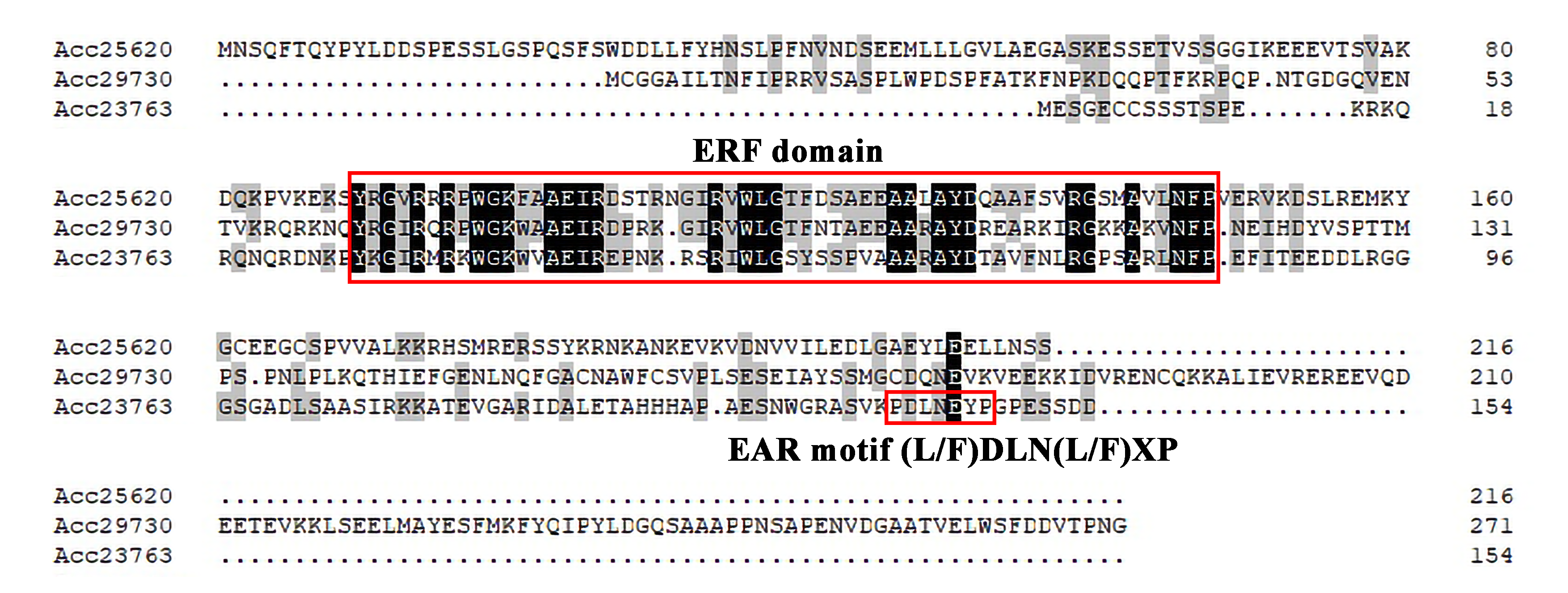

Supplement: Supplementary file 1 [file foods-10-03017-s001.zip › Figure S4.tif]

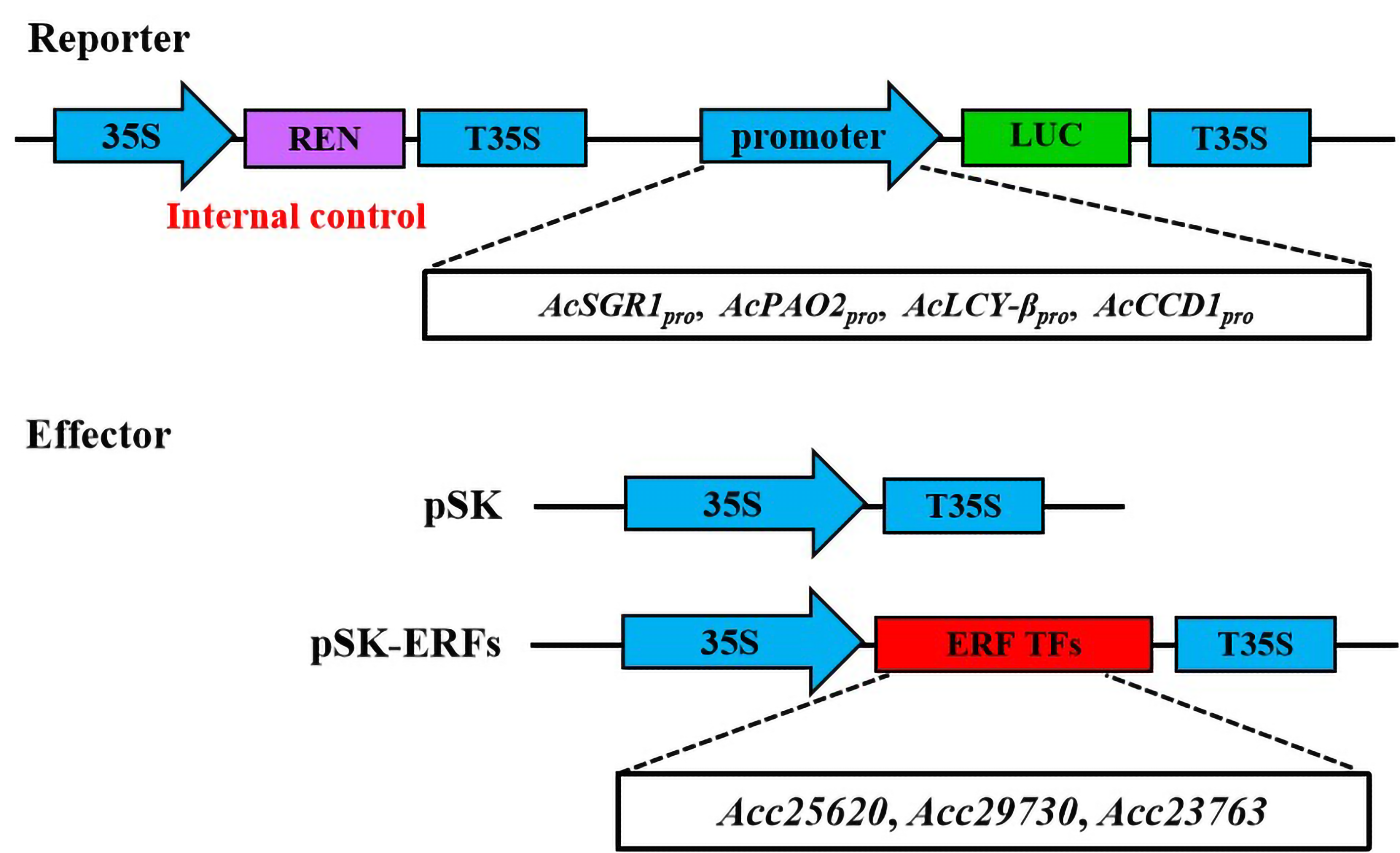

Supplement: Supplementary file 1 [file foods-10-03017-s001.zip › Figure S5.tif]
